# Identification of high risk patients with hypertrophic cardiomyopathy in a northern Greek population

**DOI:** 10.1186/1476-7120-7-37

**Published:** 2009-07-26

**Authors:** Georgios K Efthimiadis, Christodoulos Pliakos, Efstathios D Pagourelias, Despina G Parcharidou, Georgios Giannakoulas, Vasileios Kamperidis, Stavros Hadjimiltiades, Charalambos Karvounis, Stavros Gavrielidis, Ioannis H Styliadis, Georgios Parcharidis

**Affiliations:** 1 First Cardiology Department, AHEPA University Hospital, Thessaloniki, Greece

## Abstract

**Background:**

The percentage of hypertrophic cardiomyopathy (HCM) patients who are in high risk for Sudden Death (SD) constitutes only a minority of all HCM population but the incidence of SD in this subset is high (at least 5% annually). The identification of this small but important proportion of high risk HCM patients has been the clue in the clinical evaluation of these patients.

**Methods:**

Our study cohort consisted from 123 patients with HCM who are currently followed up in our Institution. Five clinical risk factors were assessed: a family history of premature SD, unexplained syncope, Non Sustained Ventricular Tachycardia (NSVT) on 24-h ECG monitoring, Abnormal Blood Pressure Response (ABPR) during upright exercise testing and Maximum left ventricular Wall Thickness (MWT) ≥30 mm. The purpose of our study was the identification of high risk HCM patients coming from Northern Greece.

**Results:**

Fifteen patients (12.2%) of the whole cohort had MWT ≥ 30 mm, 30 patients (24.4%) had an ABPR to exercise, 17 patients (13.8%) had episodes of NSVT in 24-h Holter monitoring, 17 patients (13.8%) suffered from syncope, and 8 patients (6.5%) had a positive family history of premature SD. Data analysis revealed that 74 patients (60.1%) had none risk factor. Twenty four patients (19.5%) had 1 risk factor, 17 patients (13.8%) had 2 risk factors, 4 patients (3.25%) had 3 risk factors, and 4 patients (3.25%) had 4 risk factors, while none patient had 5 risk factors. Twenty five patients (20.3%) had 2 or more risk factors.

**Conclusion:**

This study for the first time confirms that, although a 60% of patients with HCM coming from a regional Greek population are in low risk for SD, a substantial proportion (almost 20%) carries a high risk for SD justifying prophylactic therapy with amiodaron or ICD implantation.

## Background

Hypertrophic Cardiomyopathy (HCM) is a genetically transmitted cardiac disease characterized by myocardial hypertrophy in the absence of any other cause capable of producing the magnitude of hypertrophy present [1]. The incidence of HCM in general population is 0.2% [2]. The clinical spectrum of the disease is diverse ranging from an asymptomatic course throughout life to severe heart failure, stroke or sudden cardiac death [1]. HCM is the most common cause of sudden death (SD) in young people, including trained athletes [3]. The percentage of HCM patients who are in high risk for SD constitutes only a minority of all HCM population [4] but the incidence of SD in this subset is high (at least 5% annually) [5]. The identification of this small but important proportion of high risk HCM patients has been the clue in the clinical evaluation of these patients [4,6-8].

Highest risk for SD in HCM has been associated with any of the following noninvasive clinical markers [4,6,7,9-11]: prior cardiac arrest or spontaneous sustained ventricular tachycardia; family history of premature SD; syncope; nonsustained ventricular tachycardia (NSVT) on ambulatory (Holter) ECG recordings; abnormal blood pressure response (ABPR) to exercise; and extreme left ventricular (LV) hypertrophy with maximum wall thickness (MWT) ≥30 mm. Northern Greece has a population of about 3 million. People affected by HCM, in this area, are estimated to be 6.000. Concentrated epidemiologic and clinical data concerning the risk of SD and the natural history of HCM in this area are lacking. The purpose of our study was the identification of high risk HCM patients coming from Northern Greece.

## Methods

The study cohort consisted from 123 patients with HCM who are currently followed up in our Institution, and who completed all noninvasive clinical tests for risk stratification, ie, personal and family history, clinical evaluation, 12-lead ECG, transthoracic echocardiography, 24-h ECG monitoring, and symptom-limited upright exercise test. Patients with documented sustained ventricular tachycardia or out-of-hospital cardiac arrest and those who were on amiodarone at first evaluation were excluded. Patients' follow up commenced the time the first diagnosis was made even if the diagnosis preceded baseline patient evaluation in our clinic. The diagnosis of HCM was based on the echocardiographic appearance of left ventricular MWT greater or equal than 15 mm, in the absence of any other cause capable of producing such hypertrophy [12,13]. HCM was also considered present in patients with MWT 13 or 14 mm in the presence of a positive family history for HCM and/or ECG changes compatible with HCM. The patients were evaluated every 12 months, except otherwise was indicated.

### Echocardiography

Echocardiographic studies were performed using a GE Vingmed Vivid 7 system (GE Vingmed Ultrasound AS, Horten, Norway). Echocardiographic examination included M-mode, two-dimensional, pulsed- and continuous-wave Doppler echocardiography, and Tissue Doppler Imaging since the time it was available in our clinic. Segmental left ventricular hypertrophy was measured in two-dimensional echocardiography parasternal short axis views according to previous described methods [13]. Standard M-mode measurements were made according to the recommendations of the American Society of Echocardiography [14]. Basal subaortic gradient was measured by continuous wave Doppler echocardiography [15].

### Stress test

Patients underwent symptom-limited upright exercise test using the Bruce protocol in a Schiller AT-6C Stress Test Treadmill System. Blood pressure was measured using a mercury sphygmomanometer and auscultation of the Korotkoff sounds over the brachial artery at rest, every minute during exercise and for the first 3 min of recovery

### Holter monitoring

All patients underwent-24 h ECG monitoring (using various systems commercially available) while performing ordinary daily activities.

### Definitions

#### Syncope

A history of syncope was defined as one or more episodes of unexplained loss of consciousness preceding patients' first visit to our Hospital.

#### Premature sudden death

A family history of premature SD was defined as SD in one or more first-degree relatives <50 years old.

#### Non-sustained ventricular tachycardia

NSVT was defined as a run of three or more consecutive ventricular beats at a rate of ≥120 beats/min, lasting <30 s.

#### Abnormal blood pressure response

An ABPR was defined as a failure of systolic blood pressure to rise more than 20 mmHg, or a fall of systolic blood pressure, during exercise.

#### Risk factors

Five clinical risk factors were assessed: a family history of premature SD, unexplained syncope, NSVT on 24-h ECG monitoring, ABPR during upright exercise testing and left ventricular MWT ≥30 mm.

### Statistical analysis

Normality plots were tested using Kolmogorov-Smirnoff test for a p value > 0.05. Distributions of variables in the study cohort are summarized as means ± SD and as medians (IQR) for non-normally distributed data. Percentages are also reported. Most of the statistical estimates were performed using the Statistical Package for Social Sciences version 17.0 (SPSS, Chicago, Illinois).

## Results

Demographic and clinical characteristics of the 123 patients are outlined in Table 1. Patients' age at initial evaluation was 52.3 ± 15.6 years (16 to 83 years). Patients' mean age at the time of first diagnosis was 48.3 ± 16.4 (5 to 81 years), 82 patients (66.6%) were male. Nine patients (7.3%) were diagnosed as having HCM with congestive evolution. The diagnosis of congestive form was based on echocardiographic appearance of left ventricular MWT greater or equal than 15 mm with reduced ejection fraction (<50%) in the absence of any cause of secondary hypertrophy. In 3 patients the diagnosis confirmed by myocardial biopsy (extended myocardial disarray with hypertrophied myofibrils and areas of fibrosis). Coronary angiography and left heart catheterization was also performed in the remaining patients with HCM and systolic impairment. All these patients had normal coronaries without any valvulopathy more than moderate in severity. Coronary artery disease confirmed by coronary angiography was present in 6 patients (4.8%).

**Table 1:** Demographic and clinical characteristics of 123 patients with Hypertrophic Cardiomyopathy.

Age, years	52.3 ± 15.6 (16 to 83)
Age of initial diagnosis, years	48.3 ± 16.4 (5 to 81)

Male gender	82 (66.6%)

Referral patients	68 (55.8%)

Non-referral patients	55 (44.7%)

Clinical status	

Asymptomatic	56(45.5%)

Symptomatic	67 (54.5%)

Patients with NYHA class II	52 (42.2%)

Patients with NYHA class III/IV	15(12.1%)

Family history of premature SD	8 (6.5%)

Syncope	17 (13.8%)

ABPR	30 (24.4%)

NSVT	17 (13.8%)

Atrial fibrillation	

Paroxysmal	11 (8.9%)

Permanent	7 (5.7%)

Progression to Dilated Cardiomyopathy	9 (7.3%)

ECG	

Normal	12 (9.8%)

Abnormal	111 (90.2%)

Other disease	

Coronary artery disease	6 (4.8%)

Diabetes	6 (4.8%)

Hypertension	17 (13.8%)

### Echocardiographic data

The mean value of LV end-diastolic diameter was 4.32 ± 0.79 cm (range 2.77 to 7.05 cm), and the mean value of LV Ejection Fraction was 71.7 ± 13% (30 to 90%). The mean value of MWT was 2.16 ± 0.57 cm (range 1.42 to 3.87 cm) and the mean value of left atrial size was 4.09 ± 0.68 cm (range 2.24 to 5.90 cm). Twenty seven patients (21.9%) had resting subaortic gradient ≥30 mmHg (range 32 to 230 mmHg). Fifteen patients (12.2%) had maximum wall thickness ≥30 mm. The above data are outlined in Table 2.

**Table 2:** Echocardiographic features of 123 patients with Hypertrophic Cardiomyopathy

Left ventricular end diastolic diameter, cm	4.32 ± 0.79 (2.77 to 7.05)
Left ventricular maximum wall thickness, cm	2.16 ± 0.57 (1.42 to 3.87)

Left ventricular ejection fraction, %	71.7 ± 13 (30 to 90)

Left atrium size, cm	4.09 ± 0.68 (2.24 to 5.90)

Patients with basal gradient ≥30 mmHg	27 (21.9%) (range 32 to 230 mmHg).

Patients with maximum wall thickness ≥30 mm	15 (12.2%)

### Medical treatment

Sixty five percent of our patients were on medication at the initial or started after that. The patients mainly received b-blockers (47.9%). Amiodaron was started after first evaluation in our Institution. Thirty five percent were not on any medication. The above data are outlined in Table 3.

**Table 3:** Medical treatment of 123 patients with Hypertrophic Cardiomyopathy

**Medication**	80 (65%)
b-blocker	59 (47.9%)

verapamil	4 (3.2%)

disopyramide	3 (2.4%)

amiodaron	10 (8.1%)

diltiazem	5 (4%)

warfarin	9 (7.3%)

CEI/ARBs	13 (10.5%)

Statins	13 (10.5%)

Diuretics	8 (6.5%)

Digoxin	2 (1.6%)

Aspirin/clopidogrel	16 (13%)

**No Medication**	43 (35%)

### Risk Factors

**Figure 1:**
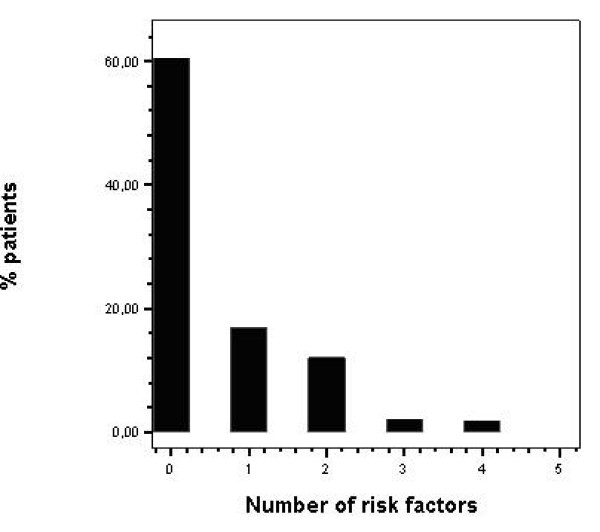
**Frequency distribution for risk factors in a Hypertrophic Cardiomyopathy population**. On the X axis is indexed the number of risk factors and on the Y axis the percent of patients in our cohort presenting this number of risk factors. Data analysis reveals that 74 patients (60.1%) had none risk factor. Twenty five patients (20.3%) had 2 or more risk factors.

Fifteen patients (12.2%) of the whole cohort had MWT ≥ 30 mm, 30 patients (24.4%) had an ABPR to exercise, 17 patients (13.8%) had episodes of NSVT in 24-h Holter monitoring, 17 patients (13.8%) suffered from syncope, and 8 patients (6.5%) had a positive family history of premature SD. Data analysis revealed that 74 patients (60.1%) had none risk factor. Twenty four patients (19.5%) had 1 risk factor, 17 patients (13.8%) had 2 risk factors, 4 patients (3.25%) had 3 risk factors, and 4 patients (3.25%) had 4 risk factors, while none patient had 5 risk factors. Twenty five patients (20.3%) had 2 or more risk factors (Figure 1).

### Follow-up

The patients were followed-up for a median period of 34.75 months (range 49.1–81.9). All patients were alive at the end of follow-up. An implantable cardioverter-defibrillator (ICD) was implanted in 10 patients for primary prevention of SD. Among these patients, 3 had 4 risk factors, 2 had 3 risk factors, 4 patients had 2 risk factors, and 1 patient had 1 risk factor. The mean follow-up period after ICD implantation, was 18.6 ± 17.5 months (1 to 48 months). Defibrillator was activated appropriately in 2 patients, by providing antitachycardia pacing for sustained ventricular tachycardia, with the restoration of sinus rhythm (The risk factors were syncope and NSVT in one patient, and syncope, NSVT, ABPR, and MWT ≥30 mm in the other patient). The annual incidence of appropriate discharges was 10.7%. One patient had an episode of inappropriate discharge (due to sinus tachycardia). Stored data were reviewed after all discharges.

## Discussion

**Figure 2:**
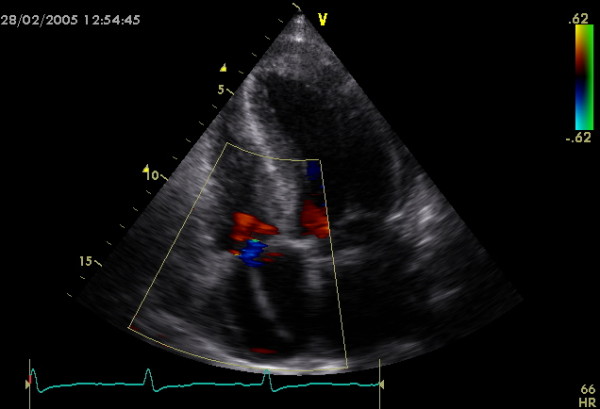
**Implantable Cardioverter Defibrillator wire in a Hypertrophic Cardiomyopathy patient**. An echocardiographic apical four chamber view showing the wire of an Implantable Cardioverter Defibrillator in the right ventricle, passing through tricuspid valve. Defibrillator was implanted in a male HCM patient presenting with previous syncope episodes and positive family history for sudden death.

It is well established that in HCM-patients with a history of sustained ventricular tachycardia or documented cardiac arrest the risk of SD is sufficiently high to warrant prophylactic therapy, irrespective of the presence of other risk factors [11,16] (Figure 2). Risk stratification in patents without a history of sustained ventricular tachycardia or documented cardiac arrest is a big clinical challenge and sometimes very difficult. Among the various clinical and echocardiographic variables considered, the simple model (based on a small number of generally accepted risk factors) proposed by Elliott et al [4] is, for the time, the most acceptable. The 5 non-invasive clinical markers indicating high risk for SD studied by Elliott et al [4] were family history of premature SD; syncope; NSVT on ambulatory (Holter) ECG recordings; ABPR to exercise; and extreme LV hypertrophy with MWT ≥30 mm. Our study group of 123 patients with HCM is unique by virtue of representing a regional cohort from a distinctive region of Northern Greece. Purpose of this study was the risk stratification of our patients, coming from a representative Northern Greek population, according to the previous referred clinical markers.

About half of our patients were referred from other centres for evaluation and treatment, and the other half was firstly diagnosed and evaluated in our clinic. As a consequence of this process, our study does not incorporate a substantial degree of patient selection bias. Our data indicates that a 60% of patients had no risk factors (55% in the study by Elliott et al [4]). A high percentage (20%) had 2 or more risk factors (12% in the study by Elliott et al [4], but in this study syncope and family history of premature SD were considered together as a single risk factor). In the study by Elliott et al [4] the presence of two or more risk factors was associated with a 4% to 5% estimated annual SD risk. Survival analysis was not performed in our study, since during the short follow-up period there was no any death among patients, and the objectives of this study was mainly the identification of the risk factors in our cohort. Although an ABPR to exercise is considered as a prognostic factor only in patients younger than 40 years of age [17], this marker was also examined in older patients, in order to collect homogeneous data in all patients. Additionally, a history of premature SD was considered positive if SD was happened before the age of 50 (<40 years in the study by Elliot et al [4]). The fact that almost 5% of patients without any risk factors experience sudden death [4], indicates that other variables, such as subaortic obstruction, atrial fibrillation and myocardial ischemia, must be taken into account in the risk stratification process.

## Conclusion

This study for the first time confirms that, although a 60% of patients with HCM coming from a regional Greek population are in low risk for SD, a substantial proportion (almost 20%) carries a high risk for SD justifying prophylactic therapy with amiodaron or ICD implantation.

## Competing interests

The authors declare that they have no competing interests.

## Authors' contributions

GKE, CP, GG and EDP conceived the original paper, collected the data, reviewed literature and wrote the manuscript. SH, IHS and GP revised the article for important intellectual content and edited the final version. GKE, DP and CK performed the ultrasounds and participated in the analysis and interpretation of data. SG and VK performed the exercise tests and participated in the interpretation of data. All authors read and approved the final manuscript.
